# Retraction: Performance and emission optimization of a CRDI engine in RCCI mode using hydrogen enriched biodiesel through grey relational analysis approach

**DOI:** 10.1371/journal.pone.0360871

**Published:** 2026-10-21

**Authors:** The PLOS One Editors

The *PLOS One* Editors retract this article [1] due to concerns about:

- Citation of unretrievable references, specifically References 3, 4, 7, 8, 9, 10, 11, 13, 14, 17, 19, 20, 21, 22, 23, 24, 25, 26, 27, 29, 30, 31, 32, 33, 34, 35, 36, 37, 38, 39, 41, 42, 43, 44, 45, 46, and 47.
- Compliance with PLOS policies on Artificial Intelligence Tools and Technologies.

We regret that the issues were not identified prior to the article’s publication.

All authors agreed with retraction.
